# Sinomenine Relieves Airway Remodeling By Inhibiting Epithelial-Mesenchymal Transition Through Downregulating TGF-β1 and Smad3 Expression *In Vitro* and *In Vivo*

**DOI:** 10.3389/fimmu.2021.736479

**Published:** 2021-11-05

**Authors:** Hongjuan He, Lihua Cao, Zheng Wang, Zhenzhen Wang, Jinxin Miao, Xiu-Min Li, Mingsan Miao

**Affiliations:** ^1^ Academy of Chinese Medical Sciences, Henan University of Chinese Medicine, Zhengzhou, China; ^2^ Microbiology and Immunology, and Department of Otolaryngology, New York Medical College, New York, NY, United States

**Keywords:** Sinomenine, airway remodeling, asthma, EMT, TGF-β1/Smad3 expression

## Abstract

Airway remodeling is associated with dysregulation of epithelial-mesenchymal transition (EMT) in patients with asthma. Sinomenine (Sin) is an effective, biologically active alkaloid that has been reported to suppress airway remodeling in mice with asthma. However, the molecular mechanisms behind this effect remain unclear. We aimed to explore the potential relationship between Sin and EMT in respiratory epithelial cells *in vitro* and *in vivo*. First, 16HBE cells were exposed to 100 μg/mL LPS and treated with 200 μg/mL Sin. Cell proliferation, migration, and wound healing assays were performed to evaluate EMT, and EMT-related markers were detected using Western blotting. Mice with OVA-induced asthma were administered 35 mg/kg or 75 mg/kg Sin. Airway inflammation and remodeling detection experiments were performed, and EMT-related factors and proteins in the TGF-β1 pathway were detected using IHC and Western blotting. We found that Sin suppressed cell migration but not proliferation in LPS-exposed 16HBE cells. Sin also inhibited MMP7, MMP9, and vimentin expression in 16HBE cells and respiratory epithelial cells from mice with asthma. Furthermore, it decreased OVA-specific IgE and IL-4 levels in serum, relieved airway remodeling, attenuated subepithelial collagen deposition, and downregulating TGF-β1and Smad3 expression in mice with asthma. Our results suggest that Sin suppresses EMT by inhibiting IL-4 and downregulating TGF-β1 and Smad3 expression.

## Introduction

Asthma, a chronic inflammatory disease of the airways, is a common heterogeneous disease that affects approximately 300 million people worldwide, resulting in 250,000 deaths per year and billions of dollars in medical expenses (1, 2). Through the involvement of various immune and structural cells, including mast cells, eosinophils, and epithelial cells, it causes pathological changes such as airway inflammation, mucus metaplasia, subepithelial fibrosis, airway hyper-responsiveness (AHR), and airway wall remodeling (3). Among these, airway inflammation and airway remodeling are the two main pathological features of asthma.

Airway remodeling refers to airway structural change that occurs in patients with asthma induced by persistent inflammation during injury and repair processes (4). It is characterized by airway wall thickening, subepithelial collagen deposition, and excessive mucus secretion (5). Airway remodeling largely causes airflow limitation and airway obstruction, endangering the lives of patients with severe asthma. Currently, most patients are well controlled and improved by well-established treatments, such as inhaled corticosteroids (ICS) and β2-adrenergic agonists. While these first-line treatments demonstrate potent anti-inflammatory effects, they are not very effective for treating airway remodeling (6). Unfortunately, approximately 10% of patients with asthma are poorly controlled, putting them at increased risk of hospitalization due to bronchial wall remodeling and airway constriction (7, 8). To prevent the progression of airway remodeling in the early stages and reduce disease severity, understanding the mechanism behind airway remodeling and identifying useful therapies are urgent (9).

Recently, it has been demonstrated that airway remodeling is associated with dysregulation of epithelial-mesenchymal transition (EMT) (10). EMT is a complex process related to tissue remodeling, in which epithelial cells gradually transform into mesenchymal-like cells through the loss of epithelial functionalities of cell-cell adhesion and polarity as well as gain of migration and invasion abilities (11, 12). During this process, biomarkers of epithelial cells, such as E-cadherin, are repressed, whereas mesenchymal markers, including vimentin, MMP7, MMP9, and alpha-smooth muscle actin (α-SMA), are upregulated. It is a novel clinical therapeutic target that is also activated in wound healing, cancer progression, and severe chronic airway diseases such as asthma and chronic obstructive pulmonary disease (COPD) (11). EMT occurs due to the stimulation of certain inflammatory factors and influence of various signaling pathways. In asthma, allergen-specific T cells are activated, and T helper type 2 (Th2) cytokines are secreted. Th17 cells are also known to modulate the disease. These cytokines interact with their receptors, activate their downstream transcription factors, increase EMT-related factors, and induce changes in airway thickness (13).

Chinese medicine has been widely used to treat bronchitis and bronchial asthma for thousands of years (14). Some traditional Chinese medicines (TCMs) have been found to inhibit airway remodeling and pulmonary fibrosis progression in asthma and COPD by targeting EMT (15, 16). Sinomenine (Sin) is an effective, biologically active alkaloid isolated from the roots and stems of the TCM Qingfengteng, also known as the climbing plant *Sinomenium acutum*. Sin has been found to demonstrate anti-inflammatory, immunosuppressive, and anti-arrhythmic effects. It was also reported to suppress collagen-induced arthritis by inhibiting Th17 factors and increasing numbers of Treg cells (17). While it has also been found to ameliorate airway remodeling in mice with asthma, the underlying molecular mechanism remains unclear (18). The aim of the present study was to explore the potential relationship between Sin and the EMT process in respiratory epithelial cells through *in vitro* and *in vivo* studies. It was found that Sin relieved airway remodeling by inhibiting EMT through downregulating the TGF-β1 and Smad3 expression.

## Materials and Methods

### Cell Culture and EMT Induction

Human bronchial epithelial cells (16HBE) were purchased from ATCC (Manassas, VA, USA) and cultured in DMEM medium (Gibco, UK) with 10% FBS (Gibco, UK) at 37°C in a humidified incubator containing 5% CO_2_. To induce EMT, the 16HBE cells were treated for 72 h with the following concentrations of LPS (Sigma-Aldrich, USA): 10, 20, 50, and 100 μg/mL. The cells were divided into four independent treatment groups: a control group, 100 μg/mL LPS group, 100 μg/mL LPS with 200 μg/mL Sin group, and 200 μg/mL Sin group.

### Cell Viability and Proliferation Assays

The cell viabilities of 16HBE cells treated with Sin were evaluated using the cell counting kit-8 (CCK8) assay (Dojindo, Japan) (19). A total of 2 ×104 16HBE cells were cultured in 96-well plates overnight until complete adherence to the walls. The cells were then incubated with Sin at final concentrations of 0, 2, 10,20, 100, 200, 500, 1000, and 2000 μg/mL for 24 h. The media were removed, and the cells were incubated with 10% CCK8 solution (Dojindo, Kyushu, Japan) for 4 h. Absorbances at 450 nm were then measured.

For proliferation assay, a total of 1*×*10^4^ cells per well were seeded in a 96-well plate, treated with the four abovementioned treatments with six replicates, and incubated for 0, 24, 48, and 72 h. The cells were then evaluated *via* CCK8 assay, as previously described.

### Wound Healing Assay

Cell migration ability was evaluated using a previously described wound healing assay (20). After growing the 16HBE cells to 90% confluence in six-well plates, the cell layers were horizontally scraped using a sterile 10 μL pipette tip across the plate. The plates were then washed with DMEM to remove debris from the straight wound area. They were then incubated with the four abovementioned independent treatments in DMEM supplemented with 2% FBS. Images of six randomly chosen fields in each wound were captured using a light microscope at 100× magnification (0 and 24 h). The migration proportion of adjacent cells to the wound area was calculated using ImageJ software.

### Migration Assay

A cell migration assay was carried out in 24-well Transwell chambers with an 8 μm pore polycarbonate membrane filter (Corning, NY, USA*)*, as described elsewhere (21). A total of 6×10^4^ 16HBE cells were cultured in the upper wells with no more than 200 μL serum-free medium. The bottom chambers were filled with 800 μL 10% FBS medium with the four abovementioned treatments. After incubation for 48 h, the migrated cells on the membrane filter were washed with phosphate buffered saline (PBS) solution, fixed with 4% paraformaldehyde for 30 min, and stained with 1% crystal violet (Amresco, Solon, OH) for 10 min. After washing and air drying, the cells were observed and photographed at 100× magnification using a microscope. The numbers of migrated cells were calculated based on six random fields per well using ImageJ software (National Institutes of Health, MD).

### OVA-Induced Asthma Model

A total of 32 six-week-old female BALB/c mice were purchased from Beijing Weitong Lihua Laboratory Animal Technology Co., Ltd. (China), fed commercial diets, and maintained under an ambient temperature of 23 ± 3°C and 12 h light/dark cycle. All animal experiments complied with the ARRIVE guidelines, “British Animal (Scientific Procedure) Act of 1986”, EU Directive 2010/63/EU, and related guidelines. All procedures described were approved by the Animal Welfare Ethics Committee of the Henan University of Chinese Medicine. The mice were randomly divided into four groups (8 mice per group): negative control, asthma, asthma with high-dose Sin (75 mg/kg), and asthma with low−dose Sin (35 mg/kg) groups.

Asthma was induced using ovalbumin (OVA, grade V, Sigma−Aldrich, USA) as previously described (9, 22). The mice were sensitized by intraperitoneal injection (i.p.) of 100 μg OVA emulsified in 2 mg Imject Alum Adjuvant (Thermo, USA) diluted in 200 μL PBS on days 0 and 7. Subsequently, the mice were intratracheally challenged with 100 μg OVA in 20 μL PBS on days 14, 17, and 20, followed by twice weekly challenges for four weeks. The control group was sensitized and challenged with PBS at the same time points. Sin was dissolved in 200 μL normal saline and intragastrically (i.g.) administered once daily from day 15–49. All mice were sacrificed 24 h after the final challenge.

### Enzyme-Linked Immunosorbent Assay

The serum was collected and centrifuged for 15 min at 12000 rpm to examine the levels of immunoglobulin E (IgE) and other cytokines. OVA-specific IgE as well as levels of IL-4, IL-6, and IL-10 in the serum were measured using a mouse IgE ELISA development kit (Mabtech, USA) and a mouse ELISA kit (Mabtech), respectively.

### Histological and Immunohistochemical Analysis

The left lung lobes were fixed in 4% paraformaldehyde and cut into 5 μm sections for histological examination and immunohistochemistry (IHC). To assess the degree of inflammation and airway remodeling, hematoxylin and eosin (H&E) staining was performed. The total area of the airway wall and the perimeter of the basement membrane (Wat/Pbm) were measured to evaluate airway remodeling (23). Masson’s trichrome staining was performed to semi-quantitatively assess subepithelial collagen deposition (1, 10). The histological analyses were performed by two independent observers.

IHC was performed as previously reported (10). Briefly, the paraffin-fixed lung sections were permeabilized with 0.02% Triton X-100 (Sigma) in PBS and then blocked with 10% normal goat serum and 2% BSA in PBS. Then, the sections were incubated with primary antibodies overnight at 4°C, with MMP9 (Abways, 1:100), MMP7 (Abways, 1:100), vimentin (Abways, 1:100), TGF*-*β1 (Immunoway, 1:100), Smad2 (Abways, 1:100), Smad3 (Abways, 1:100) and p-Smad3 (S423/S425, Abclonal, 1:100); immunized with HRP*-*conjugated goat anti-rabbit IgG secondary antibodies; and visualized with diaminobenzidine (DAB). The sections were then counterstained, dehydrated, mounted on microscope slides, and imaged under a microscope. The expression levels were semi-quantitatively scored as previously reported (21).

### Western Blotting

Total proteins from 16HBE cells or right lung tissues of mice were extracted using RIPA lysis buffer supplemented with 1% protease inhibitor (Roche Applied Science) and quantified using the BCA method. They were then diluted in 5× loading buffer, denatured, and separated *via* 10% SDS-polyacrylamide gel electrophoresis. An amount of 20 mg total protein was transferred to polyvinylidene difluoride membranes (Millipore, Billerica, MA, USA). Primary antibodies anti-MMP9 (1:1000 dilution, Abways), anti-MMP7 (1:1000 dilution, Abways), anti-vimentin (1:1000 dilution, Abways), TGF-β1 *(*Immunoway, 1:1000*)*, Smad3(1:1000 dilution, Abways), GAPDH (1:5000 dilution, Abways), and secondary goat anti-rabbit antibody Abways, 1:10000*)* were used. The images were illuminated with enhanced chemiluminescence reagent (Epizyme, Shanghai, China) and visualized using an Amersham imager 600 (GE Healthcare, Freiburg, Germany). The gray values of the protein bands were calculated using ImageJ software.

### Statistical Analysis

All experiments were performed at least in triplicate and all the values are expressed as mean ± standard error of mean (SEM). The differences among different groups were analyzed using one-way analysis of variance followed by Dunnett’s Multiple Comparison Test. If the data were not normality distribution, non-parametric multiple comparison was applied to compare differences among different groups. All statistical analyses were performed using GraphPad Prism software (version 8.0; GraphPad Software Inc., San Diego, CA, USA). Statistical significance was set at *p* < 0.05.

## Results

### Sin Inhibited Cell Migration but Not Proliferation in LPS-Exposed 16HBE Cells

**Figure 1:**
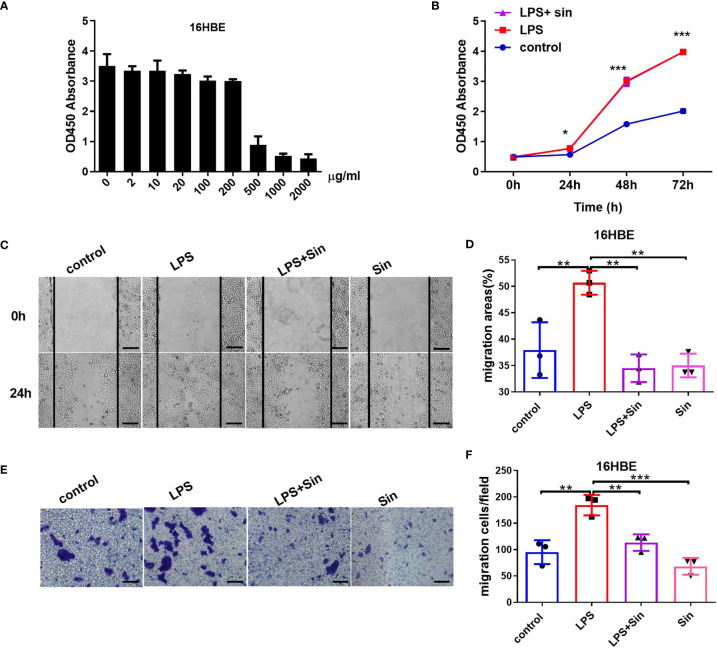
Sin inhibited cell migration but not proliferation in LPS-exposed 16HBE cells. **(A)** A total of 2×10^4^ 16HBE cells were seeded in 96-well plates and stimulated with Sin at concentrations of 0, 2, 10, 20, 100, 200, 500, 1000, 2000 μg/mL for 24 h. Cell viability was analyzed using a CCK-8 assay. The OD450 values of the cells stimulated at different doses are shown in the Y-axis. **(B)** A total of 1×10^4^ 16HBE cells were seeded in 96-well plates and divided into three groups: 100 μg/mL LPS, 100 μg/mL LPS and 200 μg/mL Sin, control (untreated) group. Cell proliferation ability was analyzed using a CCK-8 assay. The OD450 values of the cells at 0, 24, 48, and 72 h were detected and shown by the ordinate. **(C)** The cell layers were horizontally scraped using a sterile 10 μL pipette tip upon reaching 90% confluence in six-well plates. Images of the wound areas were captured at 0 and 24 h after stimulation. **(D)** Migration area was measured to analyze cell migration. **(E)** A total of 6×10^4^ HBE16 cells were seeded onto the upper wells with no more than 200 μL serum‐free medium. The bottom chamber was filled with 800 μL 10% FBS medium with 100 μg/mL LPS, 100 μg/mL LPS with 200 μg/mL Sin, 200 μg/mL Sin, or 10% FBS medium. The migrated cells were observed and captured at 100× magnification after 48 h. **(F)** Numbers of migration cells were counted from 6 randomly chosen fields. Scale bar indicates 200 μm. The data are presented as mean ± SEM from three independent experiments. The one-way analysis of variance followed by Dunnett’s Multiple Comparison Test was used. *p < 0.05. **p < 0.01. ***p < 0.001.

Airway epithelial cells are the primary targets for the inhaled environmental allergens and can produce Th2 innate cytokines to trigger allergic reactions (24). The chronic exposure of repetitive environmental injury may lead to persistent activation of pathways involved in airway epithelial repair, such as epithelial to mesenchymal transition, changes in progenitor cell migration and proliferation, and abnormal redifferentiation leading to airway remodeling (25). Lipopolysaccharide (LPS) produced by bacterial infections can exacerbate asthmatic inflammation and induce airway remodeling (26). LPS was reported to induce the proliferation, differentiation, and migration process of intestinal epithelial cell (27). In our study, we firstly suggested that LPS can promote the proliferation and migration ability of the human 16HBE cells. To explore the effect of Sin on airway remodeling, we evaluated its effect on the proliferation and migration ability induced by LPS. The viabilities of the HBE16 cells were not compromised when treated with doses of Sin less than 200 μg/mL (**Figure 1A**). The proliferation abilities of the 16HBE cells were significantly increased after stimulation with 100 μg/mL LPS. Sin did not significantly influence this effect (**Figure 1B**). However, Sin significantly inhibited LPS-induced cell motility as seen in the wound healing assay (**Figures 1C, D**). Moreover, it also inhibited LPS-induced cell migration (**Figures 1E, F**).

### Sin Inhibited EMT in LPS-Exposed Human Airway Epithelial Cells

**Figure 2:**
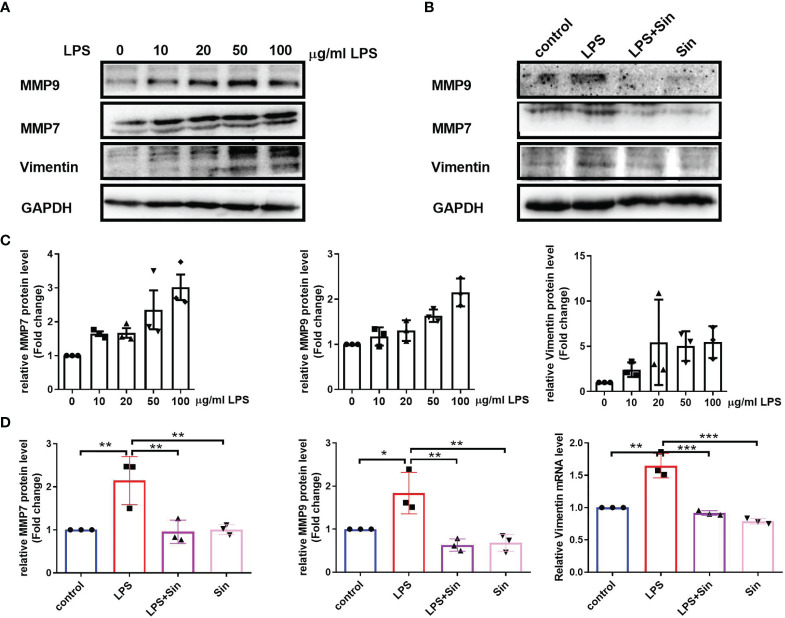
Sin inhibited EMT in LPS-exposed human airway epithelial cells. **(A)** The 16HBE cells were seeded in a 6-well plate and stimulated with LPS at concentrations of 0, 10, 20, 50, 100 μg/mL for 72 h. Protein levels of MMP9, MMP7, and vimentin were determined using Western blotting. **(B)** The 16HBE cells were seeded in a 6-well plate and divided into four groups: control (untreated), LPS (100 μg/mL), LPS with Sin (100 μg/mL and 200 μg/mL, respectively), and sin (200 μg/mL) groups. The cells were incubated for 72 h to perform Western blotting. **(C, D)** Statistical analysis of the gray values of each protein normalized to GAPDH. The data are presented as mean ± SEM from three independent experiments. The one-way analysis of variance followed by Dunnett’s Multiple Comparison Test was used. *p < 0.05. **p < 0.01. ***p < 0.001.

In order to further study the effect of Sin on EMT of 16HBE, we examined the EMT process induced by LPS exposure and detected the expression of EMT related biomarkers. Matrix metalloproteinase 9 (MMP9) was expressed by bronchial epithelium which may promote airway eosinophil infiltration and degrade the extracellular matrix (ECM)during the remodeling (18). MMP7 was identified to regulate wound repair process and was quickly upregulated after injury, which was associated with various mucosal immune processes (28). Upon evaluating EMT-related factors and MMPs at different concentrations of LPS we found that increasing concentrations of LPS increased the expression of the mesenchymal marker vimentin as well as matrix metalloproteinases MMP7 and MMP9 (**Figures 2A, C**). Additionally, Sin significantly suppressed the levels of vimentin, MMP7, and MMP9 in the 16HBE cells exposed to 100 μg/mL LPS (**Figures 2B, D**). These results suggested that Sin inhibited LPS-induced EMT in the airway epithelial cells.

### Sin Relieved Th2 Airway Inflammation in Mice With OVA-Induced

**Figure 3:**
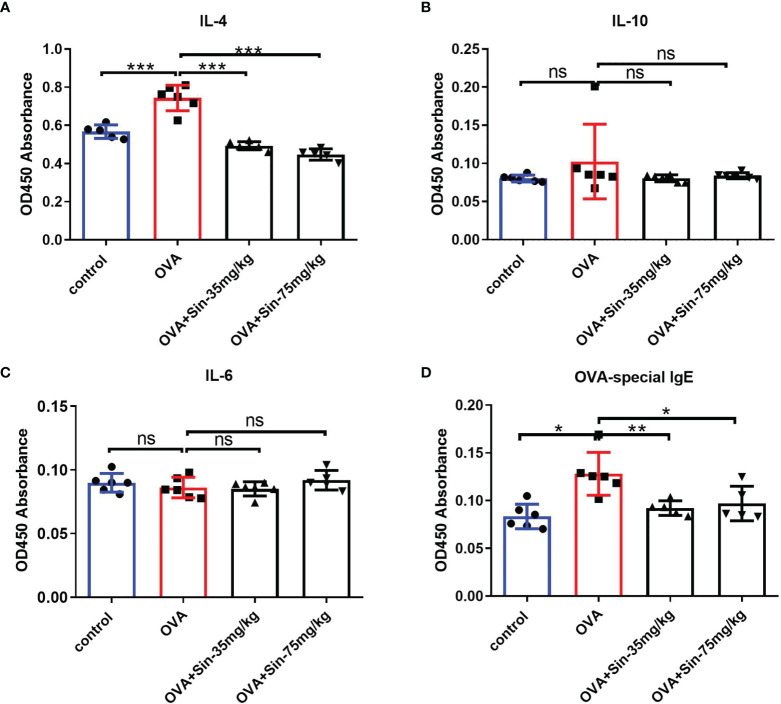
Sin relieved Th2 airway inflammation in mice with OVA-induced asthma. **(A–C)**. IL-4, IL-6, and IL-10 expressions in serum were detected using ELISA on mice from control, model, and Sin-treated (35 mg/kg or 75 mg/kg) groups. OD450 absorbances are shown in the Y-axis. **(D)** OVA-specific IgE in the serum was detected using ELISA in the abovementioned group. The data are presented as mean ± SEM (n=5-6/group). The one-way analysis of variance followed by Dunnett’s Multiple Comparison Test was used. *p < 0.05. **p < 0.01. ***p < 0.001. ns, no significant difference between the two groups.

Ovalbumin (OVA) is one of the most abundant glycoprotein allergens, which can induce IgE production and result in Th2 immune response in asthma (29). To further explore the relationship between Sin and airway remodeling, the OVA-induced asthmatic mice was established. The imbalance of Th1/Th2 is reported to cause airway inflammation −associated pathogenesis of asthma. Th2 cytokines, including interleukin−4, −5, −10, −13, were demonstrated to be involved in hyperresponsiveness and airway remodeling through activating EMT transformation (30). We detected the pharmacological effect of Sin on Th2 immune cytokines using an ELISA kit on the serum of the mice. We found that IL-4 concentrations were lower in the Sin-treated group than in the OVA group (**Figure 3A**). This indicates that Sin specifically suppressed IL-4 production. There were no significant differences in IL-10 and IL−6 concentrations (**Figures 3B, C**). Th2 allergic inflammation is classically characterized with the high levels of Th2 cytokines and immunoglobulin E (IgE) (31). In asthma patients, allergens is generally immunoreacted by high levels of IgE which was reported to mediate human allergic inflammation (32). Thus, we also examined the OVA-specific IgE level in mice. It is shown that IgE level was obviously decreased in Sin treated group compared to the OVA group (**Figure 3D**).

### Sin Relieved the Airway Remodeling in Mice With OVA-Induced Asthma

**Figure 4:**
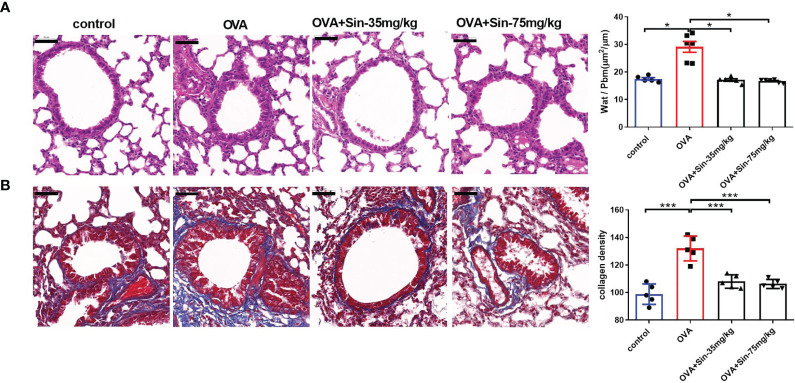
Sin relieved airway remodeling and reduced collagen deposition in the lungs of mice with OVA-induced asthma. Scale bar indicates 50 μm. **(A)** Hematoxylin & eosin (H&E)-stained lung tissue specimen sections. The total area of the airway wall and the perimeter of the basement membrane (Wat/Pbm) were used to evaluate the airway remodeling. The Wat/Pbm values (μm^2^/μm) are shown in Y-the axis. The non-parametric multiple comparison followed by Dunnett’s Multiple Comparison Test was used. **(B)** Masson staining was conducted on sections of the lung bronchi tissue, in which collagen fibers are shown as blue. The subepithelial collagen density was quantified. The one-way analysis of variance followed by Dunnett’s Multiple Comparison Test was used. The data are presented as mean ± SEM (n=5-6/group). *p < 0.05. ***p < 0.001.

Since airway remodeling is characterized by airway wall thickening, subepithelial collagen deposition, and excessive mucus secretion (5), we assessed the pathological changes caused by Sin on lung tissue sections. H&E staining revealed a significant reduction in bronchial inflammatory cell infiltration in the Sin group compared to the OVA group (**Figure 4A**). The wat/Pbm value in the OVA group was markedly increased compared to that in the control mice but obviously decreased in the treated groups. Masson staining showed that Sin remarkably relieved OVA-induced collagen deposition in asthmatic mice, as observed with the lower areas of blue-stained collagen fibers than in the OVA group (**Figure 4B**).

### Sin Suppressed EMT in Mice With OVA-Induced Asthma

**Figure 5:**
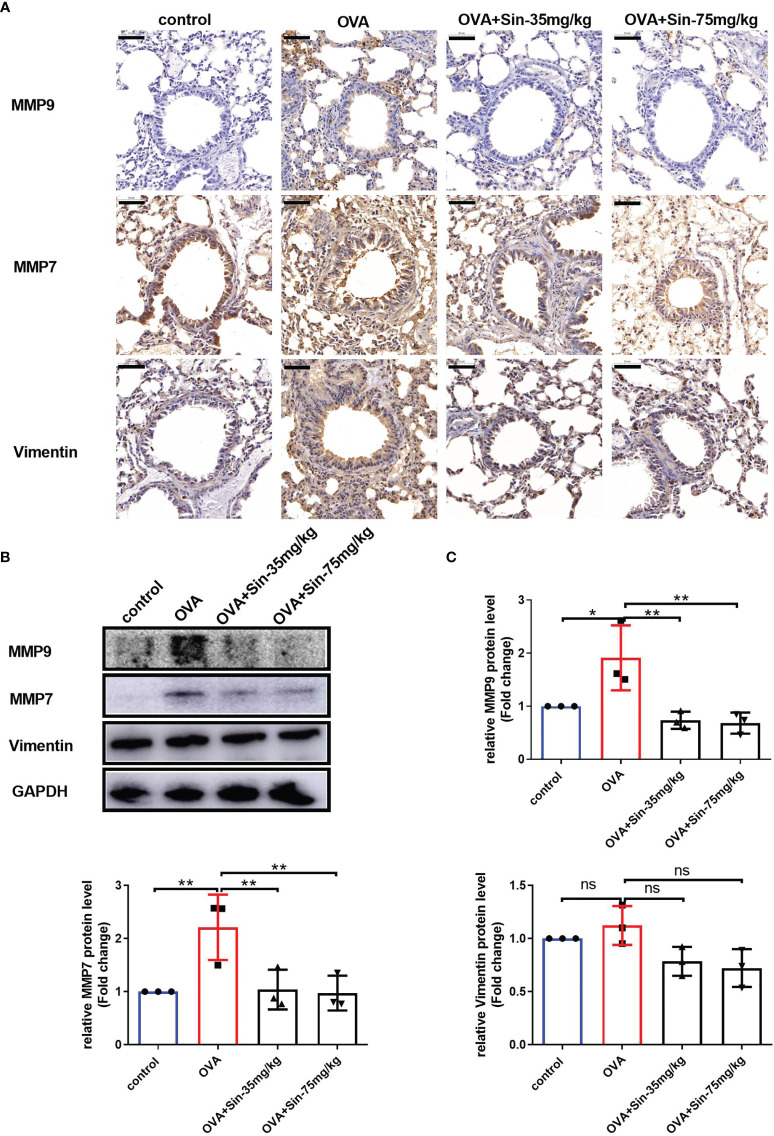
Sin reduced the EMT of the airway epithelial cells in the lungs of the mice with OVA-induced asthma. **(A)** The protein levels of MMP9, MMP7, and vimentin were detected *via* immunohistochemistry. Scale bar indicates 50 μm. **(B)** The expressions of each protein were determined using Western blotting. **(C)** Statistical analysis of the gray values of each band normalized to GAPDH. The data are presented as mean ± SEM from three independent experiments. The one-way analysis of variance followed by Dunnett’s Multiple Comparison Test was used. *p < 0.05. **p < 0.01. ns, no significant difference between the two groups.

To examine the EMT process in mice with OVA-induced asthma, we evaluated the typical indications of EMT. Immunochemical staining of the lung sections showed that the levels of vimentin, MMP7, and MMP9 in the airway epithelial cells of the Sin-treated group were significantly lower than in the OVA group (**Figure 5A**). The Western blotting results also showed that Sin obviously suppressed the expressions of MMP7 and MMP9 in the lungs of the mice with asthma (**Figures 5B, C**). These results suggested that Sin suppressed EMT in the airway epithelial cells of mice.

### Sin Suppressed the TGFβ-1 and Smad3 Expression in Airway Epithelial Cells

**Figure 6:**
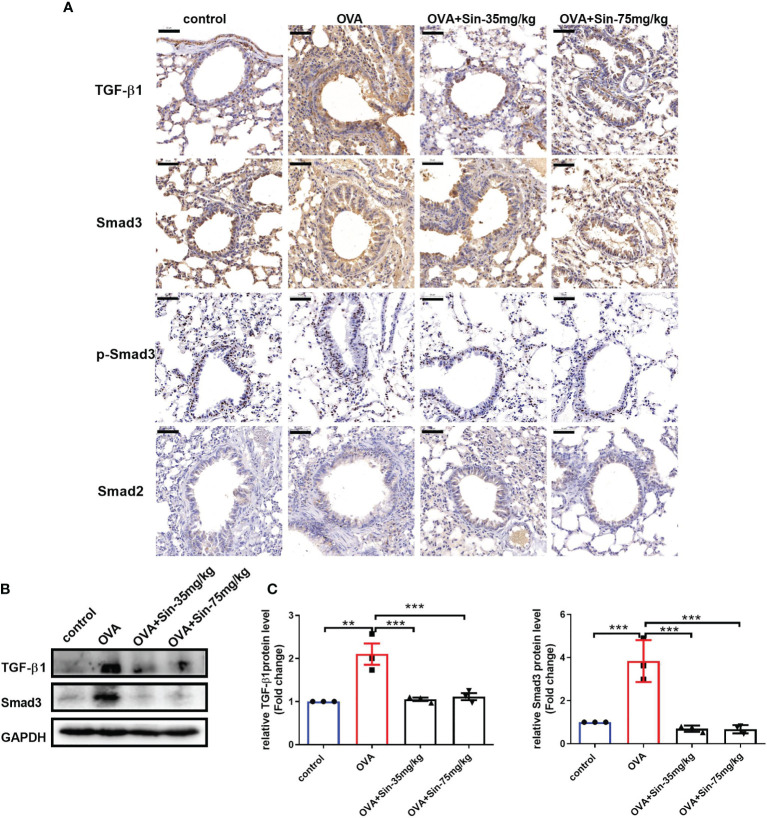
Sin inhibited the TGF-β1/Smad3 expression in the lungs of mice with OVA-induced mice asthma. **(A)** Protein levels of TGF-β1, Smad3, p-Smad3 and Smad2 were detected using immunohistochemistry. Scale bar indicates 50 μm. **(B)** Protein levels of TGF-β1 and Smad3 in the lung tissue were determined by Western blotting. **(C)**, Statistical data of the gray values of each protein normalized to GAPDH. The data are presented as mean ± SEM from three independent experiments. The one-way analysis of variance followed by Dunnett’s Multiple Comparison Test was used. **p < 0.01. ***p < 0.001. ns, no significant difference between the two groups.

To assess the mechanism underlying the relieving effects of Sin on airway remodeling, we analyzed protein levels of components of the TGF-β1/Smad pathway. We found that the protein levels of TGF-β1 and Smad3 but not Smad2 or p-Smad3 were significantly increased in the lung tissues of OVA-challenged mice compared to those in the control mice (**Figure 6A**). However, when the mice were treated with 35 mg/kg or 75 mg/kg Sin, these changes were markedly attenuated as seen in the results of the IHC (**Figure 6A**) and Western blotting (**Figures 6B, C**). These results suggested that it suppressed EMT through downregulating TGF-β1/Smad3 expression.

## Discussion

Airway remodeling is an airway structural change that is characterized by airway wall thickening, subepithelial collagen deposition, and excessive mucus secretion (5). The respiratory symptoms are largely caused by airway obstruction. Airway epithelial cells are the primary targets of inhaled environmental allergens, causing the production of Th2 innate cytokines that trigger allergic reactions (24). Chronic exposure to repetitive environmental injury may lead to persistent activation of pathways involved in airway epithelial repair, such as EMT, changes in progenitor cell migration and proliferation, and abnormal redifferentiation causing airway remodeling (25). However, it is still not clear whether environmental allergens cause the proliferation and migration of airway cells and their mechanisms. There are no reports on the effect of Sin on the changes in airway epithelial cell function caused by allergens. LPS produced by bacterial infections can exacerbate asthmatic inflammation and induce airway remodeling (26). It was also reported to induce proliferation, differentiation, and migration of intestinal epithelial cells (27). In our study, we firstly suggested that LPS can promote the proliferation and migration of human 16HBE cells. We found that Sin inhibited cell migration but not proliferation in LPS-induced 16HBE cells. These results suggest that Sin may inhibit cell migration and EMT in airway epithelial cells.

OVA, one of the most abundant glycoprotein allergens, induces IgE production and causes Th2 immune responses in patients with asthma (29). Furthermore, an imbalance in Th1/Th2 has been reported to cause airway inflammation-associated pathogenesis of asthma (30). To further explore the relationship between Sin and airway remodeling, a mouse model with OVA-induced asthma was established. We found that airway inflammatory cell infiltration was significantly increased in the lungs of the mice in this model. Additionally, severe airway remodeling and changes in collagen deposition were observed. To evaluate the therapeutic effects of Sin, we assessed the histological and morphological changes in their lungs.

Th2 allergic inflammation is classically characterized by high levels of Th2 cytokines and IgE (31). In patients with asthma, allergens generally react to high levels of IgE, causing human allergic inflammation (32). Thus, we examined the levels of Th2 cytokines and OVA-specific IgE in mice. Our results showed that Sin relieved Th2 inflammatory responses and suppressed levels of IL-4. In the human body, serum IgE concentration is very low. During an allergic response, the presence of IL-4 and IL-13 induces B cells to produce allergen-specific IgE (33). Once IgE is released into the circulation and attached to the allergic effector cells, an immediate hypersensitivity reaction occurs (34, 35). Thus, efforts to decrease IgE levels are considered very important for allergies. Furthermore, we also demonstrated that Sin regulated levels of OVA-specific IgE. Our results suggested that Sin can effectively attenuate OVA-induced airway inflammation, airway thickness, and subepithelial collagen deposition.

It is generally believed that airway remodeling is caused by chronic exposure to an inflammatory environment, leading to repeated asthma attacks. Respiratory symptoms are mainly caused by changes in airway structure. However, the mechanism of how and when airway remodeling occurs is still unclear. A previous study has reported that airway remodeling may occur in the early stages of asthma and is associated with EMT dysfunction (10). The protective role of Sin against airway inflammation has also been reported, and its role in EMT in asthma is unclear. In our OVA-induced asthma model, the mesenchymal marker, vimentin, was increased.

To explore the relationship between Sin and EMT, we evaluated EMT-related factors and matrix metalloproteinases. Matrix metalloproteinase 9 (MMP9), which is expressed in the bronchial epithelium, promotes airway eosinophil infiltration and degrades the extracellular matrix (ECM) in remodeling (18). We also found that MMP9 levels were significantly increased in lung tissue and airway models. Our results demonstrated that Sin could inhibit MMP9 expression, suggesting that Sin may suppress the thickening of the airway wall. MMP7, which is associated with various mucosal processes, was identified to regulate the wound repair process and found to be quickly upregulated after injury (28). It attenuates ciliated epithelium cell differentiation and enhances wound closure and cell migration, which are required for re-epithelialization after injury. The adhesion of cells to collagen is enhanced by MMP7 through indirect regulation of α2β1 integrin affinity (36). MMP7 has also been shown to induce EMT in various cancers (37, 38). However, the relationship between MMP7 and airway remodeling has not yet been studied. Our results showed that MMP7 expression was significantly increased in lung tissue and airway models. Moreover, MMP7 levels in the airway were significantly lower in the Sin group than in the OVA induced group. We also found that Sin significantly suppressed the levels of vimentin, MMP7, and MMP9 in LPS-induced 16HBE cells. These results indicate that Sin may inhibit collagen deposition and airway remodeling by regulating EMT.

TGF-β1 is a major inducer of EMT that is secreted by damaged or repairing epithelium. It has also been identified to be a main mediator of airway remodeling (39). Smad3 signaling is required for allergen-induced airway remodeling. It is suggested that airway remodeling is reduced in OVA-challenged Smad3-deficient mice (40). TGF-β1 induced EMT has also been implied in airway remodeling in lung allograft tissue (41).To gain insight into the mechanisms underlying the effects of Sin, we measured levels of several proteins in the TGF-β1/Smad pathway. We observed that TGF-β1 and Smad3 not Smad2 were increased in the lungs of OVA-induced mice. We also found that Sin inhibited the increases in the expressions of TGF-β1 and Smad3. Furthermore, Sin had been shown to attenuate renal fibrosis by increasing Nrf2 and mitigates profibrogenic signaling of TGF-β1/Smad (42). Sin was suggested to reduce TGF-β1-induced pSmad2/pSmad3 signaling in clear-cell renal carcinoma cells (43). p-Smad3-S423/S425 was also reported to be related to EMT. In our study, we did not observe the changes in p-Smad3, indicating that OVA may increase the expression of Smad3 without directly regulating its phosphorylation. There was also a synergic action between TGF-β1 and IL-4 in terms of induction of EMT, which can induce epithelial cells to the cell cycle together (44). IL-4 treatment leads to endogenous TGF-β1 release, which subsequently induces MMP7, MMP9, and vimentin expression (45, 46). Our results suggested that Sin may suppress MMP7, MMP9, and vimentin expression by inhibiting IL-4 and downregulating TGF-β1 and Smad3 expression.

Taken together, we demonstrated that Sin relieved airway remodeling by inhibiting EMT through downregulating TGF-β1 and Smad3 expression. However, there were some limitations to our study. Since we have not identified the direct target protein of Sin in the body, we evaluated the effects of Sin on EMT through histopathology, serology, and cell function experiments. More detailed basic and clinical studies are needed to evaluate the efficacy and mechanism of airway remodeling.

## Conclusion

Sin suppressed cell migration but not proliferation and inhibited vimentin, MMP7, and MMP9 protein expression in 16HBE cells exposed to LPS. In an OVA-induced asthma mouse model, it decreased OVA-specific IgE and IL-4 levels in the serum, relieved airway remodeling, attenuated subepithelial collagen deposition and inhibited EMT process. Our results suggest that Sin relieves airway remodeling by inhibiting EMT through the IL-4 and downregulating TGF-β1 and Smad3 expression.

## Data Availability Statement

The raw data supporting the conclusions of this article will be made available by the authors, without undue reservation.

## Ethics Statement

The animal study was reviewed and approved by the Animal Welfare Ethics Committee of the Henan University of Chinese Medicine.

## Author Contributions

Conception and design: HH and X-ML. Acquisition of data: HH, LC, and ZhengW. Analysis and interpretation of data: HH, LC, ZhengW, ZhenW, and JM. Manuscript writing: HH. Final approval of manuscript: HH, LC, ZhengW, ZhenW, JM, X-ML, and MM. All authors contributed to the article and approved the submitted version.

## Funding

This work was financially supported by grants from Henan Province Scientific and Technological Project (212102310344,202102310472), Key scientific research projects of colleges and universities in Henan Province (20A360009), and Henan University of Traditional Chinese Medicine Doctoral Research Fund (RSBSJJ2018-12).

## Conflict of Interest

The authors declare that the research was conducted in the absence of any commercial or financial relationships that could be construed as a potential conflict of interest.

## Publisher’s Note

All claims expressed in this article are solely those of the authors and do not necessarily represent those of their affiliated organizations, or those of the publisher, the editors and the reviewers. Any product that may be evaluated in this article, or claim that may be made by its manufacturer, is not guaranteed or endorsed by the publisher.
